# ‘Do they ever think about people like us?': The experiences of people with learning disabilities in England and Scotland during the COVID-19 pandemic

**DOI:** 10.1177/02610183221109147

**Published:** 2022-06-27

**Authors:** Nathaniel Scherer, Phillippa Wiseman, Nicholas Watson, Richard Brunner, Jane Cullingworth, Shaffa Hameed, CHARLOTTE PEARSON, Tom Shakespeare

**Affiliations:** 4906 London School of Hygiene & Tropical Medicine, England; 3526 University of Glasgow, Scotland; 4906 University of Strathclyde, Scotland; 4906 London School of Hygiene & Tropical Medicine, England; 3527 University of Glasgow, Scotland; 4906 London School of Hygiene & Tropical Medicine, England

**Keywords:** COVID-19, disability, health, learning disabilities, vulnerability

## Abstract

People with learning disabilities in England and Scotland have experienced an increased risk of illness and death during the COVID-19 pandemic. Drawing on data of a longitudinal qualitative study with 71 disabled people and 31 disability organisations, this article examines the experiences of 24 people with learning disabilities in England and Scotland during the pandemic, reflecting on what rendered them vulnerable and placed them at risk. Qualitative interviews were conducted with participants and key informants at two timepoints; June–August 2020 and February–April 2021. Findings emerged across four key themes: failure to plan for the needs of people with learning disabilities; the suspension and removal of social care; the impact of the pandemic on people’s everyday routines; and lack of vaccine prioritisation. The inequalities experienced by people with learning disabilities in this study are not particular to the pandemic. We explore the findings in the context of theoretical frameworks of vulnerability, including Fineman’s conceptualisation of a ‘vulnerability paradigm’. We conclude that the structured marginalisation of people with disabilities, entrenched by government action and inaction, have created and exacerbated their vulnerability. Structures, policies and action must change.

## Introduction

The COVID-19 pandemic has had a catastrophic impact on the 1.5 million people with learning disabilities in the UK. People with learning disabilities have been more likely than the general population to contract COVID-19, and more likely to experience poorer health outcomes and mortality. In 2020, people with learning disabilities in Scotland were twice as likely to have severe symptoms and three times more likely to die of COVID-19 (Henderson et al., 2021). In England, people with learning disabilities had 8.2-fold higher rates of COVID-19 related death during the first wave (March-May 2020) and 7.2-fold higher rates during the second (September 2020-February 2021) (Williamson et al., 2021). The Learning Disabilities Mortality Review (LeDeR) indicated that 35% of people with learning disabilities who died from COVID-19 lived in residential care settings, and many who died experienced difficulties accessing COVID-19 tests, learning disability nurses and other healthcare support (LeDeR Programme, 2020). Baksh et al. (2021) further exemplified the disparities in healthcare experienced by people with learning disabilities, which have been ‘magnified’ during the pandemic (Baksh et al., 2021). The authors found people with learning disabilities more likely than the general population to present with severe illness at the point of admission, corresponding to late presentation at hospital and postulated a result of poor symptom recognition, communication difficulties and exclusion from digital information and public health campaigns. Despite having more severe symptoms on admission, people with learning disabilities were less likely to access appropriate treatment, including intensive care units. Research led by Hastings and Hatton, and authored by Flynn et al. (2021), found severe reductions in social care and support for people with learning disabilities, associated with negative impacts on wellbeing and mental health, with two-thirds of their sample having experienced anger, sadness or anxiety (Flynn et al., 2021). Similar experiences for people with learning disabilities have been demonstrated internationality, with evidence emerging from the Netherlands, Ireland and Italy (Buonaguro and Bertelli, 2021; Embregts et al., 2020; McCausland et al., 2021). These experiences have renewed debate on what makes people with learning disabilities vulnerable to morbidity and mortality. In this paper, we explore this vulnerability in the context of the pandemic, with consideration of Fineman’s conceptualisation and theory.

Health and social inequalities experienced by people with learning disabilities in the UK are not new or unique to COVID-19. LeDeR has demonstrated increased rates of mortality among people with learning disabilities in England every year since 2016, with care reported to fall short of expected good practice (LeDeR Programme, 2019). Research over recent years has shown that people with learning disabilities face daily exclusion and inequality, with evidence demonstrating higher risk of abuse, discrimination, isolation, loneliness, unemployment and long-term mental health conditions (Cooper et al., 2015; Emerson and Baines, 2011; Malli et al., 2018; Wiseman and Watson, 2021). In 2008, the Joint Committee on Human Rights presented the UK government with a striking report on the human rights violations experienced by people with learning disabilities, resulting from the neglect of government policy towards their health and social care, in such a way to dehumanise them and create a system that allows abuse, discrimination and indifference, perpetuating a life of isolation, poverty and social exclusion (Joint Committee on Human Rights, 2008). The report highlighted the failure of government and public authorities to improve the lives of people with learning disabilities, stemming from limited understanding, funding and commitment to human rights. Further, in 2016, an inquiry from the United Nations Committee on the Rights of Persons with Disabilities concluded that since 2010 UK Government policies had resulted in ‘grave and systematic’ violations on the rights of disabled people, including concern on evident negative attitudes and discrimination towards people with learning disabilities, high suicide rates among this population, limited employment opportunities for people with learning disabilities, and cases in which no attempt was made to resuscitate people with learning disabilities (Committee on the Rights of Persons with Disabilities, 2016). Despite these damning reports, people with learning disabilities remain one of the most excluded groups in the UK.

The COVID-19 pandemic has presented a unique situation, in which the disadvantages and inequalities experienced by people with learning disabilities have been amplified and brought to the public’s eye. In the early stages of the pandemic, attempts to curtail the spread of the virus meant that many of their support systems had to stop, as the country tried to minimise transmission. Large numbers of people with learning disabilities require multiple and intersecting forms of support across employment, education and wellbeing, and many have complex and additional needs, requiring social care support at home or day services. Most of these services closed in March 2020, with many still not fully open towards the end of 2021. With these closures and the disproportionate impact of the virus, people with learning disabilities found themselves at risk and without formalised support (Flynn and Hatton, 2021; Shakespeare et al., 2021; Pearson et al., 2022). Evidence from the first wave of the pandemic found people with learning disabilities socially isolated and worried about lost support, lost routine, and decreased health and wellbeing (Flynn et al., 2021).

Through longitudinal qualitative research with disabled people in England and Scotland, we sought to further understand the experiences of people with learning disabilities and their families during the pandemic, including responses from national government, local authorities and social care providers. Before presenting our findings, we explore the ideas of Martha Fineman and others to discuss the concept of vulnerability among people with learning disabilities, both during and before this pandemic.

### Learning disability, vulnerability and COVID-19

Much of the debate on high mortality and morbidity from COVID-19 among people with learning disabilities has focused on vulnerability, and in particular, clinical vulnerability to the virus. Courtney and Cooper (2021), in an editorial in the British Medical Journal, drew together evidence from a range of studies to point to what they described as the ‘extreme vulnerability’ of this group to COVID-19 (Courtenay and Cooper, 2021). They argued that people with learning disabilities often have high levels of co-morbidity, many of which make them more clinically susceptible to COVID-19 and its effects. They also highlighted high levels of prejudice and discrimination within healthcare; for example, the way in which people with learning disabilities were disadvantaged in vaccine programmes.

In a rejoinder to this editorial, Hatton (2021) warned against and challenged the systemic use of the word ‘vulnerable’ in relation to people with learning disabilities and COVID-19. He argued that describing people with learning disabilities as vulnerable to the virus ‘locates the disproportionate risk of death from Covid 19 (and before Covid 19 too) as a property of individual people with learning disabilities. This not only removes agency from over a million UK citizens, but also can serve to remove any sense of urgency or even responsibility to see these inequalities for the injustices they are and to do something about them that matches the scale of the injustice.’ (Hatton, 2021).

According to Hatton, the work cited in Courtney and Cooper’s editorial focused not on clinical vulnerability, but on the way that society treats people with learning disabilities. It is this treatment, he argues, that makes people with learning disabilities ‘vulnerable’. Hatton cites, as examples, the limited support received from health and social care during the pandemic, the discrimination of healthcare practitioners, and the living arrangements of people with learning disabilities, with large numbers placed in congregate settings, a hotspot for virus transmission. It is these social influences that have created the health inequalities experienced by people with learning disabilities during the COVID-19 pandemic. Hatton expresses the concerns of many disability theorists around the use of the term vulnerability, who argue that the concept may construct those deemed vulnerable as less able, less competent and more prone to harm (Scully, 2014).

Grear (2006) and Turner (2021) argue that all human beings are corporeally vulnerable, resulting not just from human embodied frailties, but through structural processes and institutions that produce social vulnerabilities (Grear, 2006; Turner, 2021). Further, Andorno (2016) defines ‘vulnerability’ as the ways in which some groups (in particular disabled people) are likely to experience greater harms due to their identities (Andorno, 2016). From Andorno’s perspective, the purpose of understanding particular groups as vulnerable is not to construct those groups as inherently vulnerable, but rather to ensure greater protections and rights from state violence, discrimination and oppression. As such, vulnerabilisation can be understood as a social process and outcome of marginalisation, rather than being relegated to biomedical spheres and individual impairments.

We can further point to these social processes of vulnerabilisation through the work of Martha Fineman (2008) and the way she has deconstructed vulnerability to create what she termed the ‘Vulnerability Paradigm’ (Fineman, 2008). Fineman argues that focusing on acts of discrimination alone is too narrow; it produces an analysis of a particular action or moment of harm that privileges the individual and there is a danger that the ‘historical, systemic and institutional structures that surround that moment’ remain unexplored (Fineman, 2015). Equality has traditionally focused on fighting forms of discrimination with regards to race, gender, religion, etc. by providing the same treatment to all, in what Fineman has called the ‘equal protection doctrine’. Fineman argues that this understanding of equality is inadequate (Fineman, 2008). By highlighting individuals and individual actions it fails to address the impact of economic and social disadvantage on wellbeing. The equal protection doctrine does not challenge the underlying structures and practices. This is particularly harmful when applied to disability. Instead, Fineman calls for an approach that takes account of the context and structures that surround and create harm. Fineman argues that this is best achieved by focusing on differences rather than equality, by understanding the way in which different subjects are constructed in political and legal discourse. In recent years, Fineman’s concepts have been increasingly applied to disability and learning disabilities (Clough, 2017; Heikkilä et al., 2020; Scully, 2014; Snipstad, 2021).

Fineman’s approach starts from the basis that all people share common characteristics but that there are differences between them (Fineman, 2004). According to Fineman, we are all vulnerable; it is, she argues, part of the universal human condition. We are all susceptible to change and physical or social harm. Vulnerabilities are then embodied and embedded (Fineman, 2015; Fineman, 2017). Embodied differences are those that arise from biology, development, social relations or conventions. These include identity categories, such as ethnicity, gender and disability, and the way in which these have been constructed to create hierarchies and bias. People with learning disabilities are often excluded because they have been marked as incapable, inferior, weak or dangerous, for example. This exclusion is not universal; it is socially imposed, contingent and can take many forms (Scully, 2014). Vulnerabilities are also embedded in ‘social relationships and within societal institutions’ (Fineman, 2015). These economic, social, cultural and institutional relationships create embedded differences and it is these, Fineman argues, that shape our lives and create vulnerability. Emphasis is placed not on the individual, but on the structural, societal or institutional failings that cause vulnerability. People with learning disabilities are thus *made* vulnerable through social systems that exclude, actively harm and invisibilise, to render them devalued in everyday communities. These combine with and reinforce embodied vulnerabilities.

We have used Fineman’s vulnerability paradigm as an overarching concept to explore the experiences of people with learning disabilities. In our analysis we have aimed to locate the experiences of people with learning disabilities during COVID-19 within the structures and practices that have rendered them vulnerable, placing them at greater risk.

## Methods

The findings presented here are drawn from a subset of data collected as part of a longitudinal qualitative study into the experiences of disabled people in England and Scotland. In this larger study, we conducted semi-structured, in-depth interviews with 71 disabled people and 31 key informants from disability support organisations across England and Scotland (Shakespeare et al., 2021). Two rounds of interview were held with this group of participants and key informants in June–August 2020, during the first wave of the pandemic, and February–April 2021, towards the end of the second. Of the 71 participants in the larger study, 24 were people with learning disabilities, caregivers or proxy respondents, from which the findings of this article are drawn.

Ethical approval for this study was obtained from the Research Ethics Committee at the London School of Hygiene & Tropical Medicine (Ref: 21878).

### Participants

Participants were recruited through Disabled People’s Organisations (DPOs) and other third sector organisations, via online advertisements and mail-outs. Participants were purposively selected, in order to maximise variation in line with Patton’s sampling strategy, based on impairment (e.g. physical, sensory, intellectual), gender, age and geographic location (Patton, 1990).

Of the 71 disabled participants interviewed, 13 were adults with learning disabilities, six caregivers and proxy respondents of adults with learning disabilities, and five caregivers of children with learning disabilities. In total, 24 participants with learning disabilities were included (Table 1), along with 31 key informants based in organisations of and for disabled people.

**Table 1.** Sample characteristics.

Variable	Number (%)
*Participant*	
Direct	13 (54%)
Proxy (caregiver)	11 (46%)
*Location*	
England	8 (33%)
Scotland	16 (77%)
*Age*	
<18	3 (13%)
18–65	20 (83%)
>65	1 (4%)
*Gender*	
Female	13 (54%)
Male	11 (46%)
*Living arrangement before COVID-19*	
Own home	6 (25%)
Family home	13 (54%)
Residential care	5 (21%)
*Total*	24 (100%)

### Data collection

The interview guide was developed by the research team and adapted iteratively in response to participants’ engagement in the first interviews. Questions explored the experiences of disabled people and their caregivers during the pandemic across key life areas including work, education, leisure, social care, health and the government pandemic response. The key informant interview guide included similar topics of relevance to the experiences of disabled people, but also focused on organisational and sector response, including changes in the organisation’s work and member support, collaboration with government and other third sector organisations, and challenges in service provision.

### Inclusive research

Social distancing measures were in place in the UK at the time of each interview and we adjusted research practices to maintain inclusivity (Walmsley and Johnson, 2004). Participants were provided the information sheet and consent form in easier to read formats during recruitment and were invited to interview via telephone, Zoom or email, depending on their preference. People with learning disabilities are a digitally excluded group and we worked with third sector organisations to recruit those without immediate access to online technology, ensuring their inclusion. For those that found telephone, Zoom or similar online communication technology inaccessible, we offered response via email or proxy. We encouraged participants to have a supporter present for the interview if helpful to them. Some interviews were conducted with a caregiver and self-advocate together. We also invited participants to speak with us a day or two before the scheduled interview, providing an opportunity to familiarise themselves with the online technology, to ask questions about the process and to get comfortable with the research team. Researchers used Easy Read materials to support the informed consent procedures. The methods used and described were trialled across six interviews and adapted in response to participant feedback. Each interview lasted between 30–60 min. All participants received a £20 voucher for taking part and accessible research summaries have since been provided to all.

### Data analysis

Interviews were transcribed verbatim, anonymised and stored on a secure server. Each transcript was coded in NVivo 12. The research team developed an initial coding frame and adapted this as needed throughout the analysis using mechanisms of reflexive thematic analysis (Braun and Clarke, 2006). The teams at the London School of Hygiene & Tropical Medicine and the University of Glasgow cross-reviewed eight transcripts in the early stages of analysis to ensure consistent coding. During the analysis, themes were identified, reviewed and refined by the research team. Participant narratives and quotes are presented in this paper and have been anonymised through use of pseudonyms. Quotes are from people with learning disabilities, unless otherwise stated. The round of interview from which a quote is derived is noted by T1 (first round) and T2 (second round).

## Findings

Findings emerged across four key themes. First, we explore the limited inclusion of people with learning disabilities in pandemic response strategies. Second, we examine how the suspension and removal of social care impacted on people with learning disabilities and their families. Third, we discuss the impact of the pandemic on people’s everyday routines, which left many isolated. Finally, we explore the initial lack of vaccine support for people with learning disabilities and the fight for prioritisation.

### Afterthought, lack of thought or no thought

From the outset of the pandemic, respondents described how people with learning disabilities were rarely considered in the national response. They felt invisible and ignored.

When I watched them [news briefings] with mum I never once heard the word learning disability. We were completely forgotten about.

((voice breaking and close to crying)) Do they ever think about people like us? When they mention the word ‘shielding’, you think about the elderly, the vulnerable. Aren’t we vulnerable? They’re not the only ones shielding. We’re missing out on our social care and our healthcare because of the coronavirus, because of shielding. But could they care less? No. Do they care? No. (Kelly, England, T1)

Government neglect was experienced not just in the early stages when the response could perhaps be expected to be uncertain, but throughout.

I think we’ve just been pushed underneath the carpet, because I don’t think the government give a damn. As long as they’re okay and they’ve got their wage packet, they don’t give a – excuse my language here – they don’t give a toss. (Sally, Scotland, T2)

Policy focus was given to hospitals and it is clear from our data that people with learning disabilities were not considered a priority. At the root of this problem was neglect and limited understanding on both their needs and the way that they live their lives. This was seen among both national and local actors.

I inveigled myself into a meeting of [Local Authority senior management] quite recently and I was just fascinated about how little they knew about people with learning disabilities and how little they knew about people in their communities who had learning disabilities. (CEO, national learning disability organisation, Scotland, T2)

This lack of engagement and understanding is illustrated by the failure to provide information and guidance to people with learning disabilities in multiple and accessible formats.

Poppy: The government should give more information, that's what I think. They should give more information. They should get more information and I think they should explain it more to people with learning disabilities, ‘cause some people don't understand what they're saying.

Basil: No, that is the problem, isn’t it?

Poppy: That’s what they should’ve… that's what they should have done. ‘Cause I've found out that their words, that they’re using big words, not little words. They don’t break it down. (Poppy and Basil, England, T1)

As the pandemic progressed, there were attempts by governments to provide information in Easy Read format, but these were often only available online, making it difficult for digitally excluded people with learning disabilities to access. Inaccessible information resulted in misunderstanding and increased fear and anxiety, creating and deepening risk. Third sector organisations stepped in to provide accessible materials on the virus, with examples produced by Beyond Words and the Scottish Consortium for Learning Disabilities. Third sector organisations also hosted COVID-19 information sessions on Zoom and helped people with learning disabilities access the regular government briefings and other news stories.

Insufficient government consideration on the needs of people with learning disabilities impacted on the quality of care and support they received and it is to this we now turn.

### Dissolution of care and support

At the start of the pandemic, many people with learning disabilities saw their social care services and packages cut. Often social care was removed overnight and with very little warning.

[name of provider], who provide most of Maurice’s care, phoned me up and said:

‘Obviously this virus is getting more serious, so we’ve had to prioritise the support we provide, and in two days, Maurice’s care will be ceasing altogether, and it’s over to you. You need to now cover all of it.’

So as you can imagine, that was a surprise. (Pearson et al., 2022) (Abby, sister of Maurice, Scotland, T1)

For the majority of our participants, support packages were cut, with no further word from providers as the pandemic progressed. Where social workers and others did get in touch, it was often cursory and had little effect. Some told us, for example, that the only contact they received was related to their annual review. This was seen by many as very poorly timed and insensitive, after no contact for months.

The day centre called me maybe about a month ago to say it was time for his review and could they perhaps come round to do it in the garden? And I said: ‘Are you having a laugh here? You haven’t seen him for five months. What is there to review?’. And they said that: ‘We have to do it, we’ve got paperwork to do’. And I said: ‘No, I’m sorry, it’s just a tick box exercise’. (Kris, mother of Jim, Scotland, T1)

Although the governments talked often about increased payments for unpaid family carers, our respondents reported little additional funding being made available in practice, even at the second-round of interviews in 2021. In fact, many families were asked to continue paying for support, despite support being withdrawn.

The first wave of activity and the first wave of calls we received from families were about the withdrawal of social care support. In one or two instances we had a situation where some very well-known social care providers, who also are charities, not only withdrew support but asked the families to continue paying for support. Along the lines of saying to them: ‘Well we need to keep your space open for your son or daughter so it would be better if you kept paying’. And these weren’t isolated incidents, these were coming from a number of families across Scotland. (CEO, national learning disability organisation, Scotland, T2)

People also received little or no guidance from the government or local authority on how they could get support in their home, or how they could make that support COVID secure. We were told of occasions where staff who normally worked with people with learning disabilities were transferred to work in the care of older people. As the Care Manager of a service in Scotland told us, her service provided support to 50 people with learning disabilities before the pandemic. At the time of interview in June 2020, they supported only 14 people. The rest were left unsupported.

This shift of care staff meant a lack of consistency in the delivery of care. Where support was provided, the workers were often changed at short notice, putting people with learning disabilities at increased risk. Participants had to develop their own guidelines and procedures on COVID-19 measures in their home, and in many cases, the provision of personal protective equipment (PPE) for staff in the domiciliary sector was hard to source. It was also, for some, hard to persuade staff to wear PPE, and unlike in care homes and hospitals, it does not appear that guidelines were provided for domiciliary care staff. If they were, they were not well disseminated and there was limited accountability.

Participants often had to fight with local authorities to restart their support packages and some of our participants had still not seen these reinstated by early 2021. Throughout the pandemic, there was an expectation from governments, local authorities and service providers that families would be able to revert to becoming chief providers of care. For many family members this produced stress, burnout and mental health concerns, as they tried to juggle 24/7 care, with no support or respite. One mother told us of how she was accidentally copied into the minutes of a social care provider, who had discussed her request for short break care. They were going to refuse, as they believed she, as a mother, needed to be doing more. All of this despite her caring for her son 24/7 for five months, without a break.

It made me understand that there was a complete lack of awareness of caring for someone in your own home in a national lockdown, 24 hours 7 days a week and not even being able to go out to work. That’s unforgiveable. (Donna, mother of Ryan, England, T2)

Most participants expressed grave concern that cuts to social care packages would continue once the pandemic was over, as families had effectively ‘proven’ that they could undertake the role of the state, despite substantial socio-economic impact and impact on wellbeing.

I feel as well that both social and healthcare services have a very limited – I’m going to use limited – understanding of what challenges people with learning disabilities have. I think they feel that it’s like people with disabilities are making special demands… wanting special treatment. But actually that’s not really it, they just want the same outcomes as other people. They just want to live their lives. (Abby, sister of Maurice, Scotland, T2)

Ultimately, participants felt abandoned. They were asked to fend for themselves and often without the proper means. Providers were rarely in touch, despite payment for support frequently remaining in place. In their joint interview Kris, the mother of Jim, a 30-year-old man with learning disabilities who lived with her, told us how, even by March 2021, nobody had been in touch with them:

I’ve never been asked the question if it’s okay, if we’re doing okay… Nobody called me to see if I was managing without respite for a year, so from that point of view, I wouldn’t say that the local authority had looked after us. I think it’s been a case of, if we were in crisis, they would expect that we would get in touch, and because we haven’t, they’re assuming everything’s alright. (Kris, mother of Jim, Scotland, T2)

Participants told us one of the major issues throughout the pandemic was the lack of consultation with them and their families from government, local authorities and social care services about their social care needs, with most having to react as best they could to a top-down approach, which resulted in withdrawn and inadequate provision.

### Disrupted routines

The suspension of services and support disrupted daily routines. Of course, everyone’s normal routines were disrupted during the pandemic, but the impact of closures on the lives of people with learning disabilities was profound. For many of our participants, activities such as drama groups and book clubs provided structure, routine, and security, in otherwise precarious and isolated lives. Day services also acted as platforms to socialise, and many reported missing their friends and connections regularly seen at these settings. With these closed during the pandemic, many participants felt isolated and lonely, with a sense of loss and worthlessness. Some felt they had lost their independence, and now relied too much on family.

I also feel my independence has gone, due to now I am always with my parents or if we go out they are always supporting me but I’m not having my independence. Before I was out on my own and using public transport.

Also my freedom of choice is reduced as less opportunities to choose that are of interest to me.

Now I am transported by cars all of the time by my parents. It’s very hard as they changed daily depending on how I am coping.

I often feel sad no clubs and hobbies are available yet. (Frances, Scotland, T2)

These feelings of isolation and lost independence led some to show increased instances of distress; for example, through challenging behaviours, including violence towards themselves and family members.

That was really hard, you know, people were saying that they felt they had lost their children, that their child now was so low in mood and, you know, lethargic, not carrying out activities. Self-injurious behaviours, communicating in adverse ways, had put on weight, lost skills, not able to do the things that they used to be able to do. And a couple of families saying: ‘I don’t know whether I’ll ever have my son back again’. Oh, I mean, honestly it was enough to bring you to tears really. (CEO, national learning disability organisation, Scotland, T2)

Many expressed concern that without the stimulation provided by these services, including the social interaction central to activities, they would lose hard fought life skills. Caregivers were concerned that they could not provide as stimulating an environment or the variety of experiences as these services and as a consequence were seeing their loved ones regress.

It’s a real concern to, well, most of us, that it might just never reopen, and that has been suggested as a possible case scenario. That is a real worry, because as I was saying earlier, Lucy’s health and wellbeing have been impacted on, because she hasn’t been able to go to her day service. She needs to be out and about, she needs to be doing, she needs more challenge than she gets from just staying in the house all the time. (Priscilla, mother of Lucy, Scotland, T2)

With the cycle of routine broken, many were less stimulated and expressed anxiety about being able to return to their pre-pandemic routine. As the pandemic progressed, the strains and demands of lockdown started to deepen. Mental health support was, however, severely disrupted. It was removed for many, or was offered only online, inaccessible to many people with learning disabilities. One mother in England asked for mental health support from Child and Adolescent Mental Health Services (CAMHS) in March 2020, at the start of lockdown, but she did not receive a response until October 2020, with services offered only by Zoom, which was inaccessible to her son. They asked for reasonable accommodation but were told this was not possible. She paid £2000 for private mental health care instead and her son has shown substantial improvements in his wellbeing. Private mental health support is only available to a small minority of the general and learning disability population, however.

Again, third sector organisations stepped in with online activities when services closed, including online book clubs, cooking courses and quizzes, as well as mental health support lines, which were all received very positively by our participants. To overcome digital exclusion, third sector organisations provided training on how to use Zoom and some were able to provide digital tablets and laptops to those in need. That said, we were told that many people with learning disabilities remained digitally excluded throughout the pandemic.

At the second round of interviews, some had begun to see their day services and other activities resume at reduced hours, and after a few ‘teething’ issues on return, some had seen a boost to their mental wellbeing.

They were really supportive within the parameters they could work in, which for a long time was no access to the day centre whatsoever, which was hellish for my brother. And they’ve gone back to two days a week. So, he has some access then, which has given him a bit of a routine and a bit of a sense of purpose, and his mental health has levelled out a bit. So that’s helpful. (Alice, sister of Michael, Scotland, T2)

As well as social care, health management routines were also severely impacted during the pandemic. Routine health checks were suspended or delayed with consequences to the everyday health of participants. Day centre closure contributed to reduced mobility and physical health, with many of the participants missing out on organised activities, such as swimming. There were further concerns from participants with regards to foot care, dental care and diet management. Participants described how the pandemic had adversely affected the management of their epilepsy and control of their diabetes, for example.

### Delayed vaccine priority

There was also concern expressed with regards to the vaccine and protection from COVID-19. For months, people with learning disabilities were ignored for vaccine priority, despite early evidence that they had a high-risk of morbidity and mortality from the disease (Mahase, 2021; Public Health England, 2020). Self-advocates, families and third sector organisations had to fight for priority status in the vaccination programme, with a number of coordinated campaigns directed towards the government.

I have a simple example of that when I was – I’m not sure I was lobbying – but I was in a meeting with the Scottish Government’s lead for vaccination… I said: ‘So what I’m actually trying to help with here is the efficacy of your administration of your vaccination programme’. And the response I got was: ‘that would be taking somebody else’s vaccine’. And I thought, you’re not even listening to the argument, you’re not even engaging with me on this. It’s similar to that [previous] response: ‘but there’s only 8000 of them’ [people with Down Syndrome]. The human rights of people with learning disabilities have been absolutely trampled on. (CEO, national learning disability organisation, Scotland, T2)

Only after a media storm, brought into the public’s view by well-known relatives of people with learning disabilities, such as Ian Rankin and Jo Wiley, were people with learning disabilities eventually given priority. For many of our participants, their vaccination was a good experience. However, for others, there were issues. The vaccination programme prioritised people with learning disabilities registered with a GP, but people with learning disabilities frequently fall through the net and they are less visible to health services (Buszewicz et al., 2014). Many of our participants were not invited for the vaccine because they were not registered as having learning disabilities. Many had to contact their GP themselves to arrange their vaccine and many expressed confusion with the system and the eligibility criteria.

For others who received the vaccine, the experience itself was a challenge. Although people with learning disabilities had been placed in the priority group, there were often no accommodations made at vaccination centres. Information on the vaccine process was not provided in an accessible format, and often those administering had no experience working with a person with additional needs. Donna told us how her son Ryan refused the vaccine because there were no accommodations made. She asked for support from a learning disability nurse, Easy Read materials, a private space and a longer appointment. These were denied.

It's the same – there's no facility. It's a backhanded compliment, saying all these people with autism or learning disability can go and have their vaccine, but they're not going to take it up if they can't access it. It’s so cruel really. There would be uproar, wouldn’t there, with no wheelchair access to a building for vaccination. But it's exactly the same - accessibility. (Donna, mother of Ryan, England, T2)

Inaccessible vaccine procedures and delayed prioritisation increased vulnerability to the virus. Uptake of vaccinations by people with learning disabilities has been historically low (flu vaccine uptake was just 58% in 2019 to 2020) and the findings of our study reinforce the need for inclusive, targeted programmes (Public Health England, 2021).

## Discussion

Our findings leave no doubt that the pandemic and the measures introduced to stop the spread of the virus had a significant impact on the lives and wellbeing of people with learning disabilities in England and Scotland. Government action (and inaction) exposed people with learning disabilities to risk and to harm. The pandemic revealed how poorly their needs are understood and how rarely people with learning disabilities are considered in policy planning.

Based on these findings, this paper has explored how people with learning disabilities have been made vulnerable during the COVID-19 pandemic. These vulnerabilities are both embodied, in that they are underpinned by greater risk of morbidity and mortality, and also embedded in social processes and practices. Vulnerabilities are produced not just because of people with learning disabilities’ increased risk of acquiring and dying from the virus, but also because of inappropriate, inadequate and discriminatory social structures, and the processes and practices that they are embedded within. As Scully (2014) argues, vulnerabilities associated with an impairment can be ‘amplified through structural and institutional processes that distribute unequally the resources that people might use to shield themselves and foster resilience against the impact of disability’ (Scully, 2014). Our findings demonstrate the social production of vulnerability experienced by people with learning disabilities.

People with learning disabilities in England and Scotland were, initially at least, abandoned in the Westminster and Scottish governments’ response to the COVID-19 pandemic. The pandemic exposed the limited understanding and neglect of governments and other statutory agencies of the needs and lives of people with learning disabilities and their families. While the marginalisation of people with learning disabilities is not new, they have, in this pandemic, faced the same crisis in a new form. To date, almost all of the official dialogue surrounding people with learning disabilities and COVID-19 has focused on their clinical vulnerability to the virus, largely ignoring the social and cultural practices that have placed them at increased risk. As we look back over the course of the pandemic and our longitudinal data, we see a focus from government on the biomedical, with little consideration for social dimensions and the impact these have on health and wellbeing.

Our findings suggest that the marginalisation and vulnerabilisation of people with learning disabilities has been the result of government action, or rather, inaction, entrenching pre-existing inequitable social structures (Fineman, 2008; Hatton, 2021). The removal of social care during COVID-19 presents the clearest of example of this. Throughout the pandemic, social care was seen as secondary to health care; disabled people’s social care needs were neglected and restricted by the UK and Scottish governments (Pearson et al., 2022; Shakespeare et al., 2021). In a 2020 analysis of national government policy response during the first wave of the pandemic, the Health Foundation concluded that government support for social care came too late and faced widespread implementation problems (Dunn et al., 2020). This led to limited protection and support for people using adult social care, increasing unmet need for social care (Dunn et al., 2020). Removing care increased demands on people with learning disabilities and their families at a time when support was most needed, causing stress, isolation, anxiety and fear. This neglect came after years of austerity-imposed erosion in social care, which was, as noted by the Health Foundation, ‘underfunded, understaffed, undervalued and at risk of collapse’, prior to the pandemic (Dunn et al., 2020). This neglected social care, both before and during the pandemic, has deepened legacies of exclusion and disregard for the health, wellbeing and rights of people with learning disabilities (Glasby and Needham, 2020; Martinelli, 2017; Pearson et al., 2022). Further, inaccessible COVID-19 information, delayed vaccination priority, and limited care guidance for families and providers all increased vulnerabilities experienced by people with learning disabilities, impacting their wellbeing and quality of life. Our findings are consistent with other evidence generated during the pandemic; government inaction and indifference exacerbated the pre-existing vulnerabilities of people with learning disabilities during the pandemic, whilst producing new precarities, putting their lives and wellbeing at risk (Flynn et al., 2021; Armstrong and Pickering, 2020).

In September 2021, the UK government announced plans to review strategies, policies and funding for health and social care in England. In Scotland, the Feeley Review into Adult Social Care is currently under consultation (Scottish Government, 2021). If they are to have the necessary impact, these reviews must transform the existing chronically underfunded system, rather than prop up that which already exists (Pearson et al., 2022). Governments must also prioritise areas that contribute to wider health inequalities experienced by people with learning disabilities. Emerson and Hatton have identified causes of health inequalities for people with learning disabilities, from which the government must learn and act (Emerson and Hatton, 2014). Key amongst these are the social determinants of health, which include social factors such as socioeconomic disadvantage, inadequate housing, discrimination, isolation, exclusion and violence. These are central to Fineman’s model of vulnerability, and have played a key role in creating the disadvantages experienced by people with learning disabilities during the pandemic.

Entwined in this process of transformation is the need for governments to work in co-production and partnership with people with learning disabilities and their families. In part, the failure to account for people with learning disabilities during the pandemic arose because of limited consultation when planning the response, resulting in abrupt and inappropriate actions. Not only did the policies not meet the needs of this group, they served to amplify their disadvantage. People with learning disabilities were placed at increased risk because governments failed to work in partnership with them, their families and DPOs. Such engagement would have been relatively easy; there are large numbers of DPOs and other third sector organisations that could have acted as proxies at the start of the pandemic. As we discussed in an earlier article, these non-statutory agencies played a central role in covering the inaction of central government (Shakespeare et al., 2021; Cullingworth et al., 2021). DPOs across England and Scotland have called for increased funding, support and responsibility going forwards, and there must be recognition of the value they play in helping disabled people access their rights (Inclusion London, 2020; Glasgow Disability Alliance, 2020; Inclusion Scotland, 2020; Greater Manchester Disabled People's Panel, 2020).

Over the last two decades, governments across the UK have received evidence, reports and advocacy on the systematic inequalities experienced by people with learning disabilities, stemming from limited understanding, neglect and indifference (Health and Social Care Committee, 2021; Joint Committee on Human Rights, 2008; Mencap, 2007; Scior and Werner, 2015; Simmonds et al., 2018). They have had a long time to act, but have shown little willingness to do so. In December 2021, the UK Government published a follow-up report to the 2016 inquiry by the United Nations Committee on the Rights of Persons with Disabilities, covering actions in 2020 and 2021 taken in line with United Nations recommendations for welfare reform, accessible information and consultation (Department for Work and Pensions, 2021). The UK Government’s reported actions to these recommendations do not, however, reflect the information told to us by people with learning disabilities and their families, and as seen in numerous pieces of research conducted during COVID-19. Independent inquiry, review and accountability continue to be needed to ensure that governments in the UK transform the health and social landscape into one that empowers people with learning disabilities to reduce health inequalities, vulnerabilities and help them to reach their potential.

In our analysis and by employing Fineman’s vulnerability paradigm we have shown how the response of the governments has increased risk and vulnerabilised people with learning disabilities, exacerbating existing vulnerabilities and imposing new. Given this, it is not surprising that people with learning disabilities have suffered poor health outcomes and wellbeing during the COVID-19 pandemic. These inequalities are not new and urgent reform is needed; for people with learning disabilities, returning to the status quo is not an option. Governments must work in partnership with people with learning disabilities, families and disability organisations to build back a better and fairer society, addressing the challenges that contribute to poorer health, wellbeing and quality of life. In order to understand and respond to the harms that people with learning disabilities have experienced throughout the COVID-19 pandemic, we need to pay attention to the ways in which they are made vulnerable, the processes involved in making them vulnerable and the consequences on their lives.
